# Cognitive functions and anxiety among blue-collar workers in hospitals during COVID-19 pandemic

**DOI:** 10.3389/fpubh.2022.869699

**Published:** 2022-08-12

**Authors:** Abbas Mohammadi, Leila Ibrahimi Ghavamabadi, Maryam Silavi, Behzad Fouladi Dehaghi

**Affiliations:** ^1^Environmental Technologies Research Center, School of Health, Ahvaz Jundishapur University of Medical Sciences, Ahvaz, Iran; ^2^Department of Occupational Health Engineering, School of Health, Ahvaz Jundishapur University of Medical Sciences, Ahvaz, Iran; ^3^Department of Environmental Management-HSE, Ahvaz Branch, Islamic Azad University, Ahvaz, Iran

**Keywords:** coronavirus, cognitive, health, workers, hospital

## Abstract

**Background:**

The rapid spread of COVID-19 poses a major threat to public health worldwide. Hospital blue-collar workers, like other health care workers, may be under severe physical and psychological stress. This psychological distress is mainly described as symptoms of anxiety and depression, stress and cognitive function. This study aimed to investigate the effects of anxiety on cognitive function among laundry and sterilization center workers in 4 hospitals during COVID-19 pandemic.

**Method:**

This study has a cross-sectional design and it was conducted among health service workers who were occupied in health facilities (laundry and sterilization center) in 4 hospitals. In the present study, two questionnaires and a test were used to collect the data included a general health questionnaire (GHQ-28), a health anxiety index questionnaire (HAI-18) and a cognitive function test. Descriptive statistics and Spearman correlation coefficient by SPSS version 19 were used.

**Results:**

The results showed that the overall score of coronavirus anxiety in male and female workers was 38.72 ± 5.94 and 40.92 ± 4.11, respectively. The correlation between auditory attention and coronavirus anxiety is moderate and has a negative trend (*P* = 0.050, *n* = 50, and r = −0.315). Workers with higher anxiety expressed lower auditory attention. Also, correlation between visual attention and coronavirus anxiety was weak and negative (*P* = 0.032, *n* = 50, and r = −0.179).

**Conclusion:**

This study revealed that cognitive and psychological aspects of mental health can be affected by COVID-19 exposure and its due anxiety in blue-collar workers in hospitals. These findings indicate that purposeful supportive interventions should be implemented to promote workers' health and cognitive function.

## Introduction

On January 30, 2020, the World Health Organization (WHO) declared COVID-19 as an outbreak of a public health emergency and a threat to health (1). The social health-related consequences of the COVID-19 pandemic are one of the challenges of this era (2, 3). Irrational behaviors of people due to fear of being exposed to this disease are the distinguishing features of this pandemic (4). This disease has caused important mental conflicts in people, which include the seriousness of the risk of the disease, unpredictability of the situation and the uncertainty of the time of disease control (5). Social anxiety around the world is under study. The effects of an outbreak on mental health are often neglected during crisis management, while the consequences are costly (6). The increasing number of deaths and positive cases, prolonged work shifts, higher workload, inadequate personal protective equipment (PPE), media coverage, lack of specific treatment, vulnerability to infection and forced quarantine, as well as inadequate support at work, can lead to an increase in the psychological burden of health workers (7, 8). Mental health has a significant impact on individuals' performance. The effect of COVID-19 on mental health has been documented in different countries among different populations including healthcare workers (5). Providing an on-site support system for health care workers (HCW) is essential to promote their mental health (9). Anxiety, fear, depression, insomnia, and mental disorders are more common among health care workers, especially among people working in high-risk units such as the emergency room during the COVID-19 outbreak (10).

Therefore, accurate assessment of the mental health status and needs of health workers in emergencies helps managers to respond properly and reduce mental distress. Therefore, it was expected that these stressful conditions would affect mental health and potentially have long-term negative effects (11, 12). Meanwhile, the COVID-19 pandemic led to the spread of psychological stress in the healthcare staff. Mental health problems can negatively affect their attention, performance and cognitive function. Studies have shown that HCW feared the spread of the disease to their families and friends thus work conditions became tough. In addition, there are reports of high levels of symptoms of stress, anxiety and depression (13, 14). Several studies have documented high rates of anxiety and depression symptoms among health care workers during the COVID-19 pandemic with various risk factors (6, 9, 13). Anxiety and stress can produce general health problems (15). Health anxiety about COVID-19 due to ignorance and cognitive ambiguities leads to fear and overrating the risks, also lack of scientific information exacerbates this anxiety (16, 17).

Zhang et al., were among first researchers which measured healthcare workers' anxiety in Iran in early months of Covid-19 pandemic. The results showed in a population of 304 healthcare staff that 28% reported high level of anxiety, 30.6% reported depression, and 20.1% claimed distress (18). Yáñez et al., in a study on 400 healthcare workers in Peru via online survey, stated that 21.7% severe anxiety and 26.1% severe mental distress. Also, lower level of anxiety was seen in participants with higher level of education (19). Chen et al., in a survey of 252 healthcare staffs in Ecuador reported the data regarding distress and anxiety with life and job satisfaction. 28.2% showed anxiety disorders and 32.5% experienced psychological distress. Some factors such as age, work experience, taking physical exercise and marital status were also important in shaping job satisfaction in healthcare workers (20). Also, in another study by Zhang et al., 240 healthcare workers in Bolivia were examined regarding job satisfaction, life satisfaction, and turnover intention in the ongoing COVID-19 pandemic. The results showed that the participants age and their number of working days were important to predict their life and job satisfaction (21). In another study by Zhang et al., in three different countries (Peru, Ecuador, and Bolivia), online questioned 712 healthcare workers reveling that 18% experience moderate levels of anxiety, while 5% reported feeling severe anxiety. They also reported that higher organizational support is associated with lower levels of anxiety. Due to various factors which can influence the anxiety levels in healthcare workers, it was suggested to conduct similar studies in other country (22). Regarding that few studies are available on the impact of the COVID-19 pandemic on hospital workers in Iran, this study aimed to investigate the effects of anxiety on cognitive function among laundry and sterilization center workers in 4 hospitals during COVID-19 pandemic.

## Materials and methods

### Study design and study participants

This study has an observational, analytical and cross-sectional design and it was conducted among health service workers who were occupied in health facilities (laundry and sterilization center) in 4 hospitals in Iran.

The data was collected from March to June 2021. During the data collection period, Iran was experiencing the fourth and fifth waves of the COVID-19 pandemic.

Inclusion criteria: the participants are needed to have a normal general health status (physical and mental) which can be determined with the aim of the general health questionnaire.

Exclusion criteria: the participants without normal health status.

### Data collection

In the present study, two questionnaires (completed in paper and pencil method) were completed at first and were followed with the integrated visual and auditory test as follows:

### General health questionnaire (GHQ-28)

This questionnaire, developed by Goldberg, is the most well-known screening tool in psychiatry. It was validated by Taghavi in Iran (23). The questionnaire consisted of 28 questions, the questions of which include 4 subscales, each of which contains 7 questions. Questions 1–7 are related to the scale of physical symptoms and general health status (Physical Symptoms Scale included: 1. Feeling healthy and well 2. Feeling the need for tonics to get things done 3. Feeling weak and lethargic 4. Feeling sick 5. Headache 6. Feeling pressure in the head 7. Feeling hot and cold). Questions 8–14 are related to the anxiety subscale (Anxiety Symptoms Scale included: 8. Insomnia 9. Waking up in the middle of sleep 10. Feeling under pressure 11. Anger and bad temper 12. Fear and panic 13. Inability to do things 14. Anxiety), questions 15–21 are related to the scale of social dysfunction (Social Action Symptoms Scale included: 15. Ability to keep yourself busy and entertained 16. Spend more time than usual 17. Feeling good about doing things 18. Feeling satisfied with how things are done 19. Feeling useful 20. Feeling the power of decision making 21. Enjoy daily activities) and questions 22–28 are related to the scale of depression (Depression Symptoms Scale included: 22. Feeling inefficacy 23. Disappointment 24. Feeling worthless 25. Suicidal thoughts 26. Inability to do things 27. Wish to die 28. Suicide). Each question in the GHQ test has four options. The scoring method is on the Likert scale, in which the options are scored as (0–1–2–3). The overall score of each person is obtained from the sum of the scores of the four subscales. A low score on this scale indicates health and a high score indicates unhealthiness. An overall score of 23 or higher indicates a lack of general health and a score below 23 indicates mental health. The maximum score of the subject in this questionnaire will be 84.

### Health anxiety index questionnaire (HAI-18)

In this study, the short form of this questionnaire, which consists of 18 questions, was used. Each question has four options, and each option includes a description of the person's health and illness components as an item that the participant should choose one of the sentences that best describes him/her. Scoring for each sub-item is from zero to 3 points. The first option has a zero score, the second option has one score, the third option has two scores, and the fourth option has three scores. The higher the final score, the higher the health anxiety level. This questionnaire has three factors: illness, disease outcomes and general health concerns. This questionnaire was developed by Salkovskis et al., and it was validated by Nargesi et al., in Iran (24).

### Cognitive function test

The integrated visual and auditory (IVA) was used to assess auditory and visual attention on the same task (25). The IVA test was performed on an HP laptop. The participants sat in front of a laptop monitor 15 to 30 cm from the screen. A two-button mouse was placed on the right side of the laptop screen and the left button of the mouse was used to record answers. Visual stimuli (1 or 2) were presented in green, 4 cm high, for 167 milliseconds in a rectangle in the middle of a computer screen. Auditory stimuli (1 or 2) were provided with headphones and lasted 500 milliseconds. The rectangle on the computer screen was empty when presenting the audio. The result of the response of each stimulus was stored in the computer for analysis. According to the method of this test, the participant while performing his/her task is expected to react to seeing or hearing the number 1 and click on the mouse once, and if he/she sees or hears 2, he/she should not react. In this test, attention levels are assessed in both visual and auditory dimensions. Before starting the test, the ambient lighting conditions of the test place were monitored and the average illumination of 300 Lux was determined. The time duration for the test in each round was at least 8 min.

### Statistical analysis

The sample size was calculated by the formula as follows;


$$\begin{array}{l} n=\frac{N^{*}Z^{2*}p\left(1-p\right)}{d^{2}\left(N-1\right)+Z^{2*}p\left(1-p\right)} \end{array}$$

Where: Z (95%) equal to 1.96, d (5%), p is the levels of anxiety in HCWs reported in the study by Sahebi et al. (12) (25%) and N is population size (equal to 60 workers in laundry and sterilization center). The minimum sample size obtained was 50.

The descriptive analysis was performed by calculating the frequency and percentage for stratified variables and the mean and standard deviation for continuous variables. Non-parametric tests were used to analyze the relationship between potential factors related to the outcome variable. Spearman correlation coefficient test, regression analysis and 95% confidence interval (CI) were calculated.

The statistical package for the social sciences (SPSS) software for windows (version 19, IBM Inc, USA) was used for data analysis. And significance level of 5% was considered. Fifty workers from the laundry and central sterilization rooms of 4 hospitals participated in the study.

## Results

Table 1 shows the demographic characteristics of participants of the study. A total of 89.53% of participants had GHQ ≤ 23 and 56% of workers (male and female) were between 35 and 39 years old. In terms of education level, 52% had a diploma. Also, 24% of all workers are infected with COVID. The mean and standard deviation of measured parameters; mental health, coronavirus anxiety, depression, social dysfunction, physical symptoms, visual attention and auditory attention among the hospital service workers in male and female groups are presented in Table 2. The results showed that the overall score of coronavirus anxiety in male and female workers was 38.72 ± 5.94 and 40.92 ± 4.11, respectively. Mann-Whitney U test showed that there was no significant difference between the two groups. Also, the results of visual and auditory attention in two groups were 75.22 ± 20.31, 72.10 ± 18.33, 72.11 ± 18.14 and 76.18 ± 17.02, respectively. The results showed that there was no significant difference between all parameters between the two groups (Table 2). The results of the correlation between coronavirus anxiety and other factors are presented in Table 3. Spearman correlation test showed that there is a negative relationship between coronavirus anxiety and mental health (*P* = 0.040, *n* = 50 and r = −0.045). The intensity of the correlation obtained is moderate. The results showed that workers with higher mental health expressed lower coronavirus anxiety.

**Table 1:** Demographic characteristics of participants.

**Characteristic**	**Male (*n* = 40)**	**Female (*n* = 10)**
**Age range (yr)**
30–34	12 (30)	3 (30)
35–39	23 (57.5)	5 (50)
>40	5 (12.5)	2 (20)
**Job experience (yr)**
<10	8 (20)	2 (20)
11–20	28 (70)	6 (60)
>21	4 (10)	2 (20)
**Body mass index (kg/m** ^**2**^ **)**
Underweight (<18.5)	1 (2.5)	1 (10)
Normal (18.5–24.9)	24 (60)	5 (50)
Overweight (25.0–29.9)	9 (22.5)	2 (20)
Obese (≥30)	6 (15)	2 (20)
**Education**
Diploma	20 (50)	6 (60)
Technical training	19 (47.5)	3 (30)
University	1 (2.5)	1 (10)
**PPE use**
Yes	40 (100)	10 (100)
No	0	0
**Infected by COVID-19**
Yes	8 (20)	4 (10)
No	32 (80)	6 (60)

*Values are presented as number (%)*.

**Table 2:** The mean and standard deviation of measured parameters in two groups and total.

	**Male (*n* = 40)**	**Female (*n* = 10)**	**Total**	*P* ^*^
	**M ±SD**	**M ±SD**	**M ±SD**	
Mental health	43.221 ± 10.143	41.020 ± 11.021	42.122 ± 12.541	0.125
Coronavirus anxiety	38.720 ± 5.942	40.920 ± 4.111	39.924 ± 5.740	0.323
Depression	16.850 ± 6.243	18.322 ± 3.013	17.714 ± 4.140	0.118
Social function disorder	9.553 ± 5.663	7.871 ± 4.174	8.670 ± 4.476	0.340
Physical symptoms	5.521 ± 3.214	4.042 ± 3.131	4.422 ± 3.176	0.424
Visual attention	75.221 ± 20.312	72.102 ± 18.332	73.791 ± 19.694	0.083
Auditory attention	72.112 ± 18.142	76.182 ± 17.020	74.841 ± 17.323	0.087

* *P <0.05 is statistically significant*.

**Table 3:** The results of correlation between coronavirus anxiety and other variables.

		**Mental health**	**Depression**	**Social function disorder**	**Physical symptoms**	**Visual attention**	**Auditory attention**
Coronavirus anxiety	r^*^	−0.045	0.213	0.364	0.172	−0.179	−0.315
	*P* ^**^	0.040	0.050	0.041	0.012	0.032	0.050

* *Spearman correlation coefficient,*

** *P <0.05*.

According to the results, there is a weak positive correlation between coronavirus anxiety and depression (*P* = 0.050, *n* = 50, and r = 0.213). Also, social function disorder and physical symptoms showed similar results. But, the correlation between auditory attention and coronavirus anxiety is moderate and has a negative trend (*P* = 0.050, *n* = 50, and r = −0.315). On the other hand, workers with higher anxiety expressed lower auditory attention. Also, correlation between visual attention and coronavirus anxiety was weak and negative (*P* = 0.032, *n* = 50, and r = −0.179).

## Discussion

The rapid spread of COVID-19 has become a global crisis and has affected many countries, including Iran. The present study examined the employees working at laundry and sterilization service centers for hospitals and revealed that even in this working group of health care workers (although not directly exposed to COVID-19 positive patients) the mental health and anxiety was affected by the event of the pandemic. This study has focused on the anxiety issue and its related outcomes objectively. The mean anxiety score among hospital blue-collar workers in the present study was 39.92 ± 5.74. Similar findings were reported by Xiao et al., in the COVID-19 outbreak as anxiety was high among Chinese medical staff (26). Ren et al. revealed that the anxiety score among surgical nurses was 43.32 ± 9.01, and the average level of anxiety in the nurses was higher than that of the Chinese norm (27). Our results were obtained to be near these surgical nurses. Although similar studies in other groups did not show similar results (10, 28, 29). This study was performed during the 4th and 5th COVID-19 pandemic peaks in Iran. During the outbreak, the work schedule of hospital service workers became stricter and tighter. Which can be the reason for this difference in results. On the other hand, Liao et al. stated that the mean stress score was 34.64 ± 25.99 in nurses in the second line of a hospital without direct contact with positive infected COVID-19 patients expressed more stress in comparison to first-line nurses (32.70 ± 24.89) (30). Some studies revealed that anxiety had a positive correlation with stress in hospital staff (8, 26, 31). The means of visual attention and auditory attention score were 73.79 ± 19.69 and 74.84 ± 17.32, respectively, and our results showed that a negative trend between coronavirus anxiety and cognitive functions. In a study by Trabelsi et al., the results showed that physical activity during the pandemic significantly decreased and it hurts physical activity (32). Hendrickson et al. revealed that pandemic anxiety had a negative effect on mental health and performance among health care workers (33). The results of a study by Kachadourian and colleagues showed that health care workers during the COVID-19 pandemic had more feeling fatigue and lower performance (34). In another study by Wang et al., the results showed that reducing anxiety and depression in nurses leads to improving their working conditions (35). Nie et al., revealed that psychological distress in nurses during the COVID-19 outbreak was influenced by factors such as anxiety, stress and mental health (36). Similar findings are reported by this study. Turna et al. also reported that the COVID-19 pandemic has negatively affected the mental health of individuals in different jobs and social contexts (37). The results obtained by the present study mean that the participants with higher scores of mental health had lower levels of COVID-19 anxiety. Besides, the results showed that being exposed to COVID-19 infection can adversely affect the cognitive function of people including their visual attention. This finding is supported by other previous studies (38, 39). Several studies have suggested that experiencing cognitive problems can directly affect work performance, worsen feelings of anxiety, depression, and increase anxiety (40, 41). The results of the present study showed that hospital service workers due to COVID-19 anxiety also experienced cognitive outcomes in the field of visual attention and auditory attention. Therefore, protecting the mental health of these hospital staff is vital to control the pandemic and their long-term health.

This study has some limitations. The first was the small number of participants. Another limitation was that social desirability bias may affect participants' responses to the scale of anxiety and cognitive function. In such a way that they exaggerate in expressing their anxiety. Therefore, workers were asked to state the truth to minimize this kind of bias. On the other hand, regarding the pandemic conditions, the workload of these workers was much more than usual normal conditions which made them take double shifts. As a result, they had not much free time to participate in the study that is why the sample size was not in the census style.

## Conclusion

This study revealed that cognitive and psychological aspects of mental health can be affected by COVID-19 exposure and its due anxiety in blue-collar workers in hospitals. These findings indicate that purposeful supportive interventions should be implemented to promote workers' health and cognitive function. The mental health of these workers can be improved by regulating their self-efficacy and perceived social support so that coping with COVID-19 anxiety can be reached with less burden of mental problems.

## Data availability statement

The original contributions presented in the study are included in the article/supplementary material, further inquiries can be directed to the corresponding author/s.

## Ethics statement

The studies involving human participants were reviewed and approved by Ahvaz Jundishapur University of Medical Sciences (IR.AJUMS.REC.1399.767). The patients/participants provided their written informed consent to participate in this study.

## Author contributions

MS, LI, BD, and AM designed the study proposal and questionnaire and collect the research data. BD and LI shared in article writing. MS, BD, and LI analyzed the data and co-wrote the paper. All authors contributed to the article and approved the submitted version.

## Funding

This work was supported by Ahvaz Jundishapur University of Medical Sciences (grant number: U-99298).

## Conflict of interest

The authors declare that the research was conducted in the absence of any commercial or financial relationships that could be construed as a potential conflict of interest.

## Publisher's note

All claims expressed in this article are solely those of the authors and do not necessarily represent those of their affiliated organizations, or those of the publisher, the editors and the reviewers. Any product that may be evaluated in this article, or claim that may be made by its manufacturer, is not guaranteed or endorsed by the publisher.
